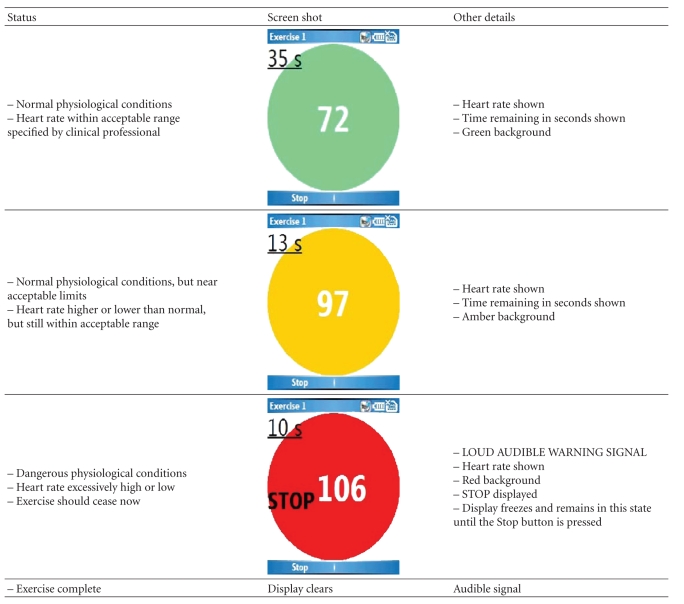# Use of a Smartphone for Improved Self-Management of Pulmonary Rehabilitation

**DOI:** 10.1155/2008/753064

**Published:** 2008-07-03

**Authors:** A. Marshall, O. Medvedev, A. Antonov

**Affiliations:** ^1^School of Computing, University of Leeds, Leeds LS2 9JT, UK; ^2^Faculty of Basic Medicine, Lomonosov Moscow State University, Moscow 119192, Russia

## Abstract

Patients suffering from chronic respiratory disease need to follow a rehabilitative exercise programme, in order to self-manage their illness and improve quality of life. Adherence to the programme is highly dependent on professional support from a physiotherapist and hence declines when patients seek to self-manage in the home. A number of requirements were identified for a Smartphone-based application in which patients are supported remotely and given automatic feedback during exercise. An application is described which will improve adherence during pulmonary rehabilitation.

## 1. INTRODUCTION

 One of the most serious challenges in healthcare today is how to support chronic disease patients to manage their lifestyle and/or therapeutic programme. Chronic diseases are on the increase [1]. Current healthcare policy considers that it is more effective clinically, economically, and socially for healthcare services to support self-management in the home, than to bring patients into hospital or clinics for regular treatment [2–4]. Self-management may involve adhering to drug therapy or another intervention, but also normally requires lifestyle changes, particularly exercise or dietary changes and in some cases cessation of smoking, alcohol consumption, and so forth [5]. Appropriate support has to be provided by community health workers, such as nurses, physiotherapists, and occupational therapists. The Smartphone application described in this article provides a tool to be used by both patients and community health workers to improve this process.

 One of the major chronic illnesses today is chronic obstructive pulmonary disease (COPD), which affects an estimated 210 million people worldwide. According to the World Health Organisation's latest estimates (2007), COPD will become the fourth most significant cause of death worldwide by 2030. In 2005, 3 million died from COPD [6]. COPD patients suffer breathlessness, shortness of breath, and chronic cough. If patients suffer an acute episode, which includes a hospital admission, they are usually advised to join a rehabilitation programme, in which they are trained to carry out a daily exercise programme. If they adhere to the exercise programme, they should improve their health significantly. The difficulty arises once they are discharged from the supervised exercise stage and expected to continue the programme at home, within their normal lifestyle [7].

We propose a support system in which the reduced level of the community health worker's time can be maximised in value, using wireless communications and personalised computing platforms. A number of telemedicine or e-health applications for respiratory medicine have already been developed and piloted [8–12]. Most pilot systems use personal home computers or provide portable devices to health workers. A system using mobile phone technology has been developed to early commercial stage, using peak flow monitors to support the self-management of asthma [8, 9]. Chronic care of COPD has been found to be made more effective by provision of 24-hour telephone support and a web-based patient self-management module [12]. The system described here is slightly different in emphasising patient motivation and management, rather than focusing on monitoring.

## 2. SMART PHONE PULMONARY REHABILITATION SYSTEM

A pilot application has been developed, based on a standard rehabilitation programme used within Wakefield Primary Care Trust, within the UK National Health Service. Patients are trained to undertake a series of 12 gentle exercises, each typically for a period of 5 minutes, per day.

### 2.1. User requirements

A stakeholder analysis was conducted to develop an understanding of the problems and issues, which the Smartphone application should address. This involved several discussions with a community physiotherapist, community respiratory nurse, and public health physician, which took place at different stages of development of the application, using an earlier demonstration version of the programme. Consultations with approximately 15 patients were conducted indirectly through the physiotherapist, who described the application in general terms and by asked for feedback on the current regime. From this, a set of user requirements were developed and are summarised in Table 1.

One issue that was identified was lack of confidence by patients in the safety of exercising. Many patients became concerned that they were short of breath, although they were actually in a completely safe state. When exercising under supervision of the physiotherapist, monitoring equipment could be connected to show that their heart rate was stable. The use of home monitoring during exercise was, therefore, identified as a desirable feature of the Smartphone application.

It was also found that motivation was a key issue in adherence to a home exercise programme, which is supported by findings reported by Calverley [5] who reports that rehabilitation programmes, whilst effective at all stages of the disease, are most successful if patients are well motivated.

### 2.2. Elements of the Smartphone application

The application has been developed using Microsoft Visual Studio for a Windows Mobile Smartphone and has been deployed on a Qtek 8300 and an O_2_ Graphite XDA phone for testing purposes. The monitoring sensor is a Nonin 4100 Bluetooth Pulse Oximeter [13]. The sensor is connected to the Smartphone by Bluetooth, prior to starting the application. The connection and pairing functions within the Smartphone operating system are used. Once this has been done the application must be launched.

The design goal for the rehabilitation application was to simplify the main user routines, which will be used on a daily basis, and provide more complex functions in separate menus. The user routines and menus are described in more detail in Section 2.3.

 Daily exercise results (including times and durations of exercise, physiological data) are saved to the phone memory card. Data is sampled every 30 seconds, recording the heart rate and the blood oxygen saturation (SpO_2_). The Nonin 4100 Bluetooth sensor sends 5 bytes of data 75 times per second. In this application, data is only sampled twice per second, and for a typical exercise period of 12 exercises each of 1–5 minutes duration, the total file size is 100–200 kB. The manufacturer's claimed accuracy is ±2 digits for SpO_2_ and ±3% for heart rate. In this application, it is the relative change of the data that is important, with absolute figures not being required to a high accuracy. Only the heart rate data is shown to the patient, as this is more variable and directly related to the rate of exercising. The blood oxygen saturation is kept for reference by the clinical users, but is generally constant throughout the exercise session. The data can be transferred using the mobile networks on a daily basis, or by data cable to a local laptop. The latter procedure, although less automated, was preferred for the population under study as it could be done weekly by the physiotherapist during a home visit. Many chronic COPD patients have limited computer skills and hence find this procedure preferable.

Clinical professionals are able to examine the data in Microsoft Excel or in a bespoke PC application, linked to patient records.

**Figure 1:**
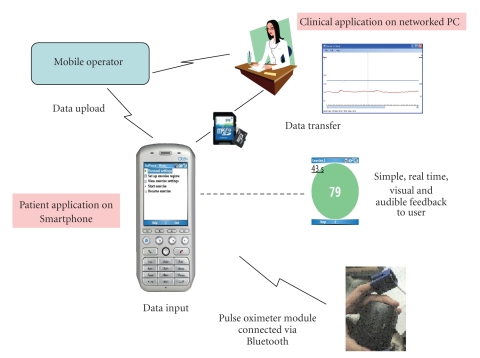
Elements of the Smartphone application.

These elements are summarised in Figure 1, which shows the main components and data transfer processes.

### 2.3. User feedback and interactivity

**Figure 2:**
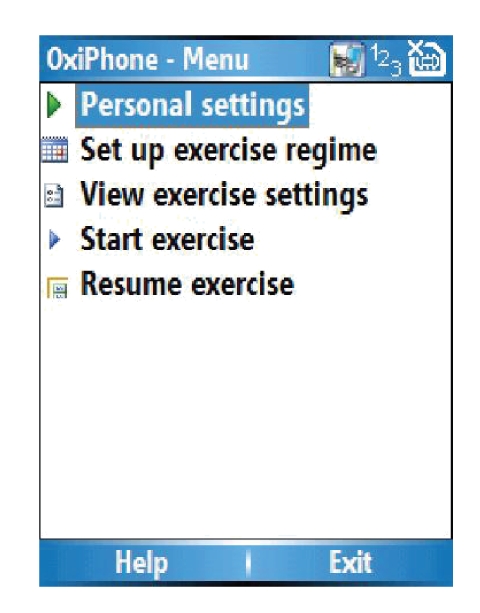
The main menu.

Users enter the application to a simple main menu, shown in Figure 2, and will normally select “Start exercise,” to go directly to exercise mode. They may also select “Resume exercise” if they had taken a break between exercises. They are then advised to connect the pulse oximeter and to start exercising. During exercise, the application provides users with real-time feedback, summarised in Table 2. The heart rate and time remaining are displayed in real time, with a green/amber/red background to denote normal/borderline/danger (STOP). In addition, audible signals are provided. A loud siren-like sound is emitted if the patient's heart rate exceeds safe limits. A different signal is emitted at the end of each individual exercise period (normally 1–5 minutes).

**Figure 3:**
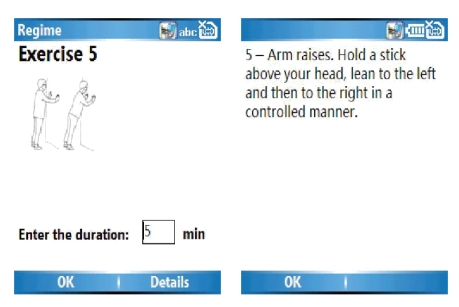
Information screens for users.

Users may also access information screens, from the main menu, to see diagrams and text describing the exercise to be done (see Figure 3).

### 2.4. Configuration of the application

**Figure 4:**
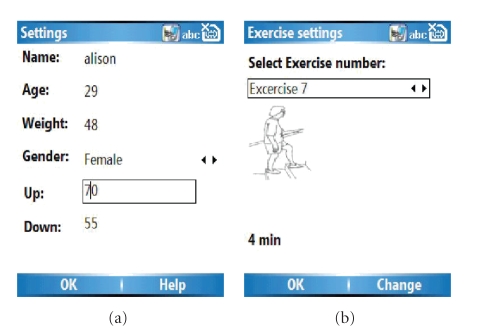
Screen shots to show configuration of application to individual user.

 The application may be configured to set the acceptable limits for the individual, in terms of minimum and maximum heart rates. The first item on the main menu, personal settings, should be selected. Basic data is recorded, as shown in Figure 4(a). The up setting and the down setting determine the limits for the feedback screen. If the user's heart rate increases to within 10% of the “Up” figure, an amber signal will be shown. When it reaches this figure, the red signal plus the alarm sound will be activated. Similarly, if the user's heart rate falls to the “Down” figure, signals will be given in the same way. The length of each exercise may also be configured, through “Setup exercise regime” and edited in “View exercise settings” (see Figure 4(b)). Configuration would normally be done by the physiotherapist or community health worker, but could be done by the users themselves.

## 3. DISCUSSION AND CONCLUSION

The pulmonary rehabilitation Smartphone application has been designed for a very specific exercise programme undertaken by chronic COPD patients. The objectives were to provide users with a tool to reduce dependence on the conventional, labour intensive services and to enable improved management of their rehabilitation programme through better self-involvement. In other words, we seek to provide a “personal physiotherapist” who is always available. The application could easily be adapted to other patient groups, who need to undertake a regular exercise programme, such as stroke patients. The use of a mobile device platform enables users to leave the home and to use a technology which is familiar. This offers significant advantages over PC-based systems, to which many individuals do not have sufficient access or proficiency to be comfortable in using for healthcare monitoring. A full patient trial has not yet been undertaken. In the UK, it is not permissible to allow patients to evaluate a new device at any level prior to approval from a local ethics committee, which has not yet been obtained. Patients were consulted indirectly via the physiotherapist, who was able to describe the application and ask questions about their views on the current rehabilitation procedure. A full user assessment and clinical evaluation are currently being planned.

## Figures and Tables

**Table 1:** Stakeholder/user requirements.

COPD patient	– Reminder to do exercises
	– Convenient (does not interfere excessively with daily lifestyle)
	– Easy to use
	– Feedback available from physiotherapist
	– Confidence that exercise activity is safe

Physiotherapist	– Ability to see if patients are adhering to programme
	– Can provide care in a shorter time than conventionally
	– Easy to use, convenient

Health service	– Reduced number of acute episodes due to failure of adherence to exercise programme
	– Management information to evaluate and monitor community care programme

**Table 2:** Feedback to users during exercise programme.